# Differentiation between active myocarditis and dilative cardiomyopathy in new-onset systolic heart failure: Potential role of T1 and T2 mapping cardiovascular magnetic resonance

**DOI:** 10.1186/1532-429X-16-S1-P289

**Published:** 2014-01-16

**Authors:** Sebastian Bohnen, Ulf K Radunski, Gunnar Lund, Katharina Koopmann, Lukas Radziwolek, Christian Stehning, Gerhard Adam, Stefan Blankenberg, Kai Muellerleile

**Affiliations:** 1 Cardiology, University Heart Center, Hamburg, Germany; 2 Radiology, University Medical Center, Hamburg, Germany; 3 Philips Research, Hamburg, Germany

## Background

There is an overlapping phenotype in patients with new-onset systolic heart failure due to myocarditis and dilative cardiomyopathy (DCM). Differentiation between both etiologies has important therapeutic and prognostic implications and is currently based on endomyocardial biopsy (EMB). In contrast, current cardiovascular magnetic resonance (CMR) techniques are of limited value in these patients with predominantly diffuse myocardial injury. This study evaluated the performance of quantitative T1- and T2-mapping CMR to differentiate between active myocarditis and DCM in patients with new-onset systolic heart failure.

## Methods

This study included 26 patients with new-onset systolic heart failure and reduced left ventricular (LV) ejection fraction (32 ± 12 %), who underwent EMB and CMR at 1.5 Tesla. The protocol included conventional sequences (T2w-STIR), Early- (T1w-TSE) and Late-Gadolinium-Enhancement (PSIR). Furthermore, T1 quantification was performed using the modified Look-Locker inversion-recovery (MOLLI) sequence before (native) and 15 minutes after administration of 0.075 mmol/kg Gadolinium-BOPTA. T2 quantification was performed using a free-breathing, navigator-gated multi-echo sequence. Native T1, post-contrast T1, extracellular volume (ECV) and T2 maps were calculated with a dedicated plug-in written for the OsiriX software.

## Results

EMB revealed variants of active myocarditis in 13 (50 %) and DCM in the remaining 13 (50 %) patients. The ROC areas-under-the-curve (AUC) to discriminate between both etiologies was 0.54 (p = 0.77) for Early-Gadolinium-Enhancement, 0.63 (p = 0.23) for Late-Gadolinium-Enhancement, 0.68 (p = 0.11) for T2w-STIR, 0.66 (p = 0.17) for native global myocardial T1, 0.72 (p = 0.05) for post-contrast global myocardial T1, 0.61 (p = 0.36) for global myocardial ECV and 0.74 (p < 0.05) for global myocardial T2. Sensitivity and specificity were 50 % and 83 % to discriminate between myocarditis and DCM using global myocardial T2 (optimal cut-off ≥ 60 ms).

## Conclusions

Assessing myocardial inflammation by T2-mapping CMR could be helpful to discriminate between active myocarditis and DCM in patients with new-onset systolic heart failure. T2-mapping CMR appears to be superior compared to conventional techniques but also to T1-mapping CMR in this context.

## Funding

No funding.

